# Genetic underpinnings of YMRS and MADRS scores variations in a bipolar sample

**DOI:** 10.1192/j.eurpsy.2023.836

**Published:** 2023-07-19

**Authors:** M. Calabrò, C. Crisafulli, A. Drago

**Affiliations:** 1 Department of Biomedical and Dental Sciences and Morphofunctional Imaging, University of Messina, Messina, Italy; 2 Unit for Psychiatric Research, Aalborg University Hospital, Aalborg, Denmark

## Abstract

**Introduction:**

Bipolar disorder (BD) is a chronic hereditary disorder. Trial and error principles and long period of untreated disorder mandate further research. Relatively recent advances in statistical computing and techniques introduced Polygenic risk scores (PRS) as predictors of the genetic susceptibility to diseases. Although they provide an estimate of the risk of developing specific pathologies, they are a genome-wide measure. PRS do not provide specific information on the biological meaning of the variants. The use of subsets of risk variants (limited to one or few related biological pathways) to calculate pathway-PRS (pPRS) may provide an estimate of the functioning of specific molecular cascades.

**Objectives:**

In the present study we calculated pPRS and tested them as potential predictive factors which, together with other clinical/environmental features, may estimate the treatment outcome of BD individuals in a clinical realistic treatment environment.

**Methods:**

1538 BD (41.39+/-12.66 years, 59.17% females) individuals from STEP-BD were included in the analysis. A latent class analysis identified three groups of patients according to the YMRS and MADRS scores variations during ˜ 1 year (308.47+/-293.83 days YMRS, 357.78+/-367.76 days MADRS). A GWAS analysis with clinical covariates provided the input for pPRS calculation. SNPs with best nominal significance and biologic relevance were prioritized through GTEx. A molecular pathway analysis (MPA) based on the interaction network of drugs used for treatment provided the genetic data needed for pPRS calculation. A Neural network was built using pPRS as features together with other variables (including Sex, Age, Scores at baseline) to predict the 3 groups previously identified. Performance was evaluated through 5-fold cross-validation, Python, R and Bash served for environments. Gene Ontology, ReactomePA and Bioconductor were key packages together with Cytoscape, Plink, impute and gtool.

**Results:**

Ten biological networks were retrieved from MPA: 1)GO:0016705 + GO:0016641, 2)GO:0019585, 3)GO:003018, 4)GO:0099589 + GO:1904014, 5)GO:0015464 + GO:1905144, 6)GO:0004935 + GO:0004364 + GO:00031690, 7)GO:1903351 + GO:1903350, 8)GO:0016917 + GO:0007214, 9)GO:0008066 + GO:0007215, 10)GO:0048016. Risk variants within the genes contained in each group were used to compute pPRS. The ten pPRS were used to compute a neural network to predict treatment outcomes.

**Conclusions:**

BD treatment is influenced by socio-demographic, clinical and genetic factors. To tackle this complexity, we tried to implement an approach where the multivariate analysis encompasses clinical analysis and the biologic background of treatment response. As a result, we can infer through a hypothesis-free approach potential pathways whose alterations may estimate treatment. At the time of writing the analyses are still undergoing, the final results will be presented and discussed at the congress.

**Disclosure of Interest:**

None Declared

